# Evaluation of a brief anti-stigma campaign in Cambridge: do short-term campaigns work?

**DOI:** 10.1186/1471-2458-10-339

**Published:** 2010-06-14

**Authors:** Sara Evans-Lacko, Jillian London, Kirsty Little, Claire Henderson, Graham Thornicroft

**Affiliations:** 1 King's College London, Institute of Psychiatry, Health Services and Population Research Department, Box P029, De Crespigny Park, London SE5 8AF, UK

## Abstract

**Background:**

In view of the high costs of mass-media campaigns, it is important to understand whether it is possible for a media campaign to have significant population effects over a short period of time. This paper explores this question specifically in reference to stigma and discrimination against people with mental health problems using the *Time to Change *Cambridge anti-stigma campaign as an example.

**Methods:**

410 face-to-face interviews were performed pre, during and post campaign activity to assess campaign awareness and mental health-related knowledge, attitudes and behaviours.

**Results:**

Although campaign awareness was not sustained following campaign activity, significant and sustained shifts occurred for mental health-related knowledge items. Specifically, there was a 24% (p < 0.001) increase in persons agreeing with the statement: *If a friend had a mental health problem, I know what advice to give them to get professional help*, following the campaign. Additionally, for the statement: *Medication can be an effective treatment for people with mental health problems*, there was a 10% rise (p = 0.05) in the proportion of interviewees responding 'agree' or 'strongly agree' following the campaign. These changes, however, were not evident for attitudinal or behaviour related questions.

**Conclusions:**

Although these results only reflect the impact of one small scale campaign, these preliminary findings suggest several considerations for mass-media campaign development and evaluation strategies such as: (1) Aiming to influence outcomes pertaining to knowledge in the short term; (2) Planning realistic and targeted outcomes over the short, medium and long term during sustained campaigns; and (3) Monitoring indirect campaign effects such as social discourse or other social networking/contact in the evaluation.

## Background

In January 2009, *Time to Change *launched a national social marketing campaign aiming to reduce stigma and discrimination against people with mental health problems[1-3]. The key campaign messages are: 'There is something you can do to help'; 'Mental illness is one of our last taboos'; and 'Mental illness is far more common than you think'. Preceding the launch of the national campaign, a smaller scale local campaign took place in Cambridge, England over a 4-week period in 2008. Compared with the subsequent national campaign, the Cambridge intervention was more localised, shorter in duration (4 weeks vs. 3 years) and had fewer financial resources (£55,000/$91,000 vs. £8.1 million/$13.4 million). Specific campaign activities in Cambridge included: advertising at bus stops, on the local radio and in the local paper; advertising using beer mats and postcards; street art in the city centre; 'talking points' around town including public sofas staffed by people with experience of mental health problems; and a one day 5-a-side football tournament.

While it might be expected that social marketing campaigns need both high intensity and long duration of exposure in order to maximise their impact, there is little evidence for this. Indeed, the effect of campaign intensity and duration may be complicated by length of follow-up period or events occurring in the social/political context which coincide with the campaign. Consequently, the results of such longer term programmes are often unclear and the evidence available regarding type of effects associated with longer duration or how to optimise campaign duration is mixed [4,5]. A Cochrane review of mass media interventions for smoking cessation, for example, found no significant association between campaign duration and effectiveness [4] and other related research has shown that serial mass-media campaigns may risk becoming redundant [6]. Other studies, however, have detected an association between longer duration and higher intensity campaigns with improved behavioural outcomes such as reductions in smoking prevalence, when compared to single interventions or short term versus long term campaigns [7,8].

Therefore, and especially in view of the high costs of social marketing and advertising, it is important to understand whether it is possible to have a significant and sustained positive effect on stigma and discrimination against people with mental health problems over a short period of time. Specifically, this study addresses the following questions, using the *Time to Change *Cambridge pilot as an example: (1) Can a short term campaign play any role in positively influencing stigma and discrimination; and (2) What types of change are possible over the short term?

## Methods

To examine these questions, face-to-face interviews were performed on separate groups of individuals pre (n = 92), during (n = 198) and post-campaign (n = 120) activity on a weekly rolling basis (i.e., approximately 50 interviews per week over an 8-week period) by trained interviewers from a contracted market research agency. The sample was restricted to the campaign target population (i.e., residents of Cambridge, aged 25-45 and of middle income socioeconomic groups B, C1 and C2) and were recruited via a market research panel. Quotas were set for gender, age, and socioeconomic group to ensure even distributions of these characteristics at each time point. Additionally, 50% of confirmed press readers were included to ensure a substantial proportion had an opportunity to see the campaign. Data were weighted for gender, age, and socioeconomic group to match the characteristics of the target population residing in Cambridge, and sampling weights were used in all analyses. Sample characteristics are shown in table 1.

**Table 1:** Characteristics of Participants in Pre and Post Campaign

	Pre-campaign n(%) n = 92	During-campaign n(%) n = 198	Post-campaign n(%) n = 120
Age			
25-29	26(28.3)	63(31.8)	35(29.2)
30-34	25(27.2)	44(22.3)	31(25.8)
35-39	17(18.5)	46(23.2)	27(22.5)
40-44	24(26.1)	45(22.7)	27(22.5)
			
Gender			
Male	43(46.7)	102(51.5)	66(55.0)
			
Employment			
Full or Part time	77(83.7)	153(77.3)	93(77.5)
Student	10(10.9)	35(17.7)	20(16.7)
Not working	5 (5.4)	10(5.0)	7 (5.8)
			
Marital			
Married/living with partner	58(63.0)	117(59.1)	73(60.8)
Single	34(37.0)	81(40.9)	47(39.2)
			
Ethnicity			
White	88(95.6)	192(97.0)	109(90.8)
Black	1 (1.1)	1(0.5)	4 (3.3)
Asian	2 (2.2)	5(2.5)	5 (4.2)
Other	1 (1.1)	0(0.0)	2 (1.7)
			
Social Contact (i.e., knowing someone with a mental illness)			
Know Someone	55(59.8)	123(62.1)	58(48.3)
No one known	37(40.2)	75(37.9)	62(51.7)

### Measures Used

The National Institute for Health and Clinical Excellence (NICE) emphasises the importance of including knowledge, attitude and behavioural components when developing and evaluating interventions aimed at behaviour change among individuals or populations. Therefore, in addition to measuring prompted campaign awareness, our evaluation included outcome measures of mental health-related knowledge, attitudes and intended behaviour. Knowledge was measured by the Mental Health Knowledge Schedule [MAKS]). The MAKS includes 6 items which assess stigma-related mental health knowledge. Overall test-retest reliability of the MAKS is 0.71 and the overall internal consistency among items is 0.65. However, since the MAKS was not developed to function as a scale the internal consistency value is less important [9]. Attitudes were assessed using 3 hypothesised items from the Community Attitudes towards Mental Illness [CAMI] scale. Of note, these 3 items were chosen to be in line with campaign targets and were chosen in collaboration with the campaign developers) [10]. Specifically these items assess attitudes regarding commonality (Virtually anyone can become mentally ill); dangerousness (People with mental health problems are far less of a danger than most people suppose); and responsibility (People with mental health problems should not be given any responsibility). Although the entire CAMI scale was not used for the campaign evaluation, the 3 items chosen come from the social restrictiveness and authoritarianism subscales. The reliability of the social restrictiveness subscale is 0.80 and the authoritarianism subscale is 0.68. Intended behaviour (the level of intended future contact with people with mental health problems) was measured by the Reported and Intended Behaviour Scale (RIBS). We specifically assessed changes in 4 intended behaviour outcomes (domains included: living with, working with, living nearby and continuing a relationship with someone with a mental health problem). which were derived from the Star Social Distance Scale. Overall test-retest reliability of the RIBS is 0.75. The overall internal consistency of the scale is 0.85 [Evans-Lacko S, Rose D, Little K, Rhydderch D, Henderson C, Thornicroft G: Development and Psychometric Properties of the Reported and Intended Behaviour Scale (RIBS), submitted]. Analyses were carried out using Stata version 10 and SAS version 9.1. This study was classified as exempt by the King's College London, Psychiatry, Nursing and Midwifery Research Ethics Subcommittee.

## Results

Overall, low to moderate levels of campaign awareness were reached. Shifts in the proportion of people reporting any campaign awareness, however, changed rapidly. Our evaluation showed that prompted campaign awareness increased incrementally over the 4-week period of the campaign and peaked during the last week of the campaign at 23%. Campaign awareness, however, fell sharply to 6% two weeks following the campaign. Notably, campaign awareness was 5% before the campaign began, indicating no change in prompted campaign awareness before and after campaign activity.

**Figure 1:**
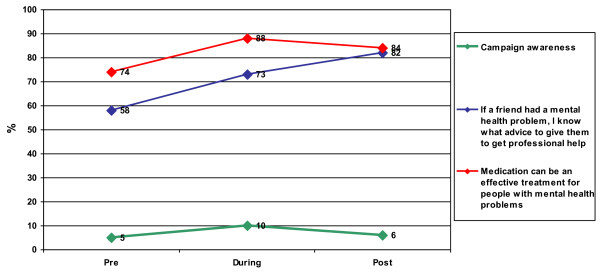
**Changes in Campaign Awareness and Significant Knowledge Outcomes Pre, During and Post Campaign Activity**.

Despite significant decreases in campaign awareness following the campaign, significant shifts did occur among participants for two out of six specific mental health knowledge-related items and these shifts were sustained following campaign activity. Specifically, there was a 24% (pre = 58%, post = 82% p < 0.001) increase in persons agreeing slightly or strongly with the statement: *If a friend had a mental health problem, I know what advice to give them to get professional help*, following the campaign. Additionally, for the statement: *Medication can be an effective treatment for people with mental health problems*, there was a 10% rise (pre = 74%, post = 84% p = 0.05) in the proportion of interviewees responding 'agree' or 'strongly agree' following the campaign (See Table 2). These differences remained significant after controlling for social contact (i.e., knowing someone with a mental illness) (p < 0.05). These changes, however, were not evident for overall MAKS score, or attitudinal or behaviour related questions based on the CAMI and RIBS (See Table 3, Figure 1).

**Table 2:** Frequencies and Percents of Participants Agreeing (Strongly or Slightly) to Correct Response Among MAKS Items

	Pre-campaign n = 92 n(%) 95% CI	Post-campaign n = 120 n(%) 95% CI
1. Most people with mental health problems want to have paid employment (T)	69(75.0) [68, 81]	90(75.0) [69, 81]
2. Most people with mental health problems go to their doctor to get help (F)	20(21.7) [16, 29]	11(9.2) [6,14]
3. Medication can be an effective treatment for people with mental health problems (T)	68(73.9) [66, 80]	101(84.2) [79, 89]
4. Psychotherapy (e.g., talking therapy or counselling) can be an effective treatment for people with mental health problems (T)	79(85.9) [80, 91]	100(83.3) [78, 88]
5. People with severe mental health problems can fully recover (T)	59(64.1) [56, 71]	72(60.0) [53, 67]
6. If a friend had a mental health problem, I know what advice to give them to get professional help (T)	53(57.6) [50, 65]	98(81.7) [76, 87]

**Table 3:** Frequencies and Percents of Participants Agreeing (Strongly or Slightly) to RIBS Intended Behaviour Items

	Pre-campaign n = 92 n(%) 95% CI	Post-campaign %Agree n = 120 n(%) 95% CI
In the future, I would be willing to **live with **someone with a mental health problem	55(59.8) [52, 67]	76(63.3) [56, 70]
In the future, I would be willing to **work with **someone with a mental health problem	73(79.4) [72, 85]	105(87.5) [82, 91]
In the future, I would be willing to **live nearby **to someone with a mental health problem	80(87.0) [81, 91]	105(87.5) [82, 91]
In the future, I would be willing to continue a **relationship with a friend **who developed a mental health problem	77(83.7) [77, 89]	90(75.0) [69, 81]

## Discussion

### Implications for campaign development and evaluation

#### Importance of Knowledge Based Outcomes

Although these results only reflect the impact of one small scale campaign, these preliminary findings suggest several considerations for mass-media campaign development and evaluation strategies. First, the data suggest that it may be easier to influence outcomes pertaining to knowledge in the short term rather than aiming to influence attitudes or behaviour. Although there may be more extensive barriers to shifting attitude or behavioural outcomes, changing knowledge around what people can do is an important outcome in itself. Much has been written as to whether improved knowledge is a useful outcome and whether public education itself is sufficient or even useful [11]. Although it is possible that some types of knowledge, such as being able to identify certain psychiatric diagnoses, may not reduce stigma or discrimination [12], there is evidence that knowledge about sources of help, or ways of helping others with mental health problems, may positively influence stigma and discrimination. Several studies of the Mental Health First Aid program, which teaches members of the public skills in how to assist people developing mental disorders, have shown improvements in stigma related outcomes such as decreased social distance from people with mental disorders, increased confidence in providing help to someone with a mental disorder, and increased help provided to others [13-17].

#### Mechanism of Change

The disparity between sustained campaign awareness and sustained changes in other campaign outcomes is informative when thinking about campaign evaluation and for exploring the mechanism of action of population level change. For instance, it is possible that in the case of the *Time to Change *Cambridge campaign, this difference exists because the campaign messages are easier to recall than actual campaign activity. Alternatively, it is possible that the campaign works indirectly to influence stigma and discrimination at the population level. For instance, one of the aims of *Time to Change *was to promote discussion about mental health problems facilitated by "talking points" around the town which included public sofas and publicity in pubs and cafes. These points were designed to facilitate discussion about or disclosure of mental health problems. Although many individuals may not have come into contact with the campaign and therefore could not recall actual campaign activity, it is possible that they were influenced by individuals who were campaign aware and who discussed the campaign messages with them. Individuals for which the campaign was more salient (e.g. mental health service users or carers) may have been motivated to champion the cause following the campaign. These 'champions' may have discussed or shared campaign messages with others who were not campaign aware. Given the significance of social contact in other studies [18,19], media campaigns may benefit from targeting individuals who could act as conduits of change to facilitate discussion, social contact or other activities.

#### Limitations

While this study contributes new and important information which may inform the planning of future media campaigns, there are limitations which should be considered. Although the sample was weighted to be representative of the target population in Cambridge in terms of gender, age, and socioeconomic group, the participants were only a sample of Cambridge and may not represent the entire target population in Cambridge. Additionally, although the study used quotas to ensure equality of the target characteristics (gender, age and socioeconomic group), different individuals were interviewed at each time point. Although this reduces the potential of an effect resulting from repeating the interview, we cannot be sure that differences in attitudes were not due to sampling characteristics. Finally, although the study included 50% of confirmed press readers to allow participants a greater opportunity to see the campaign and hence examine the relationship between awareness and attitude change, we cannot extrapolate the awareness figures to the entire population.

## Conclusions

Our evaluation demonstrated the potential of short term campaigns for achieving positive mental health-related knowledge outcomes of the type that have been shown to positively influence stigma related outcomes following a 4-week mass-media campaign. This is in line with social marketing literature which advocates targeting small and incremental changes in outcomes over an extended period of time [20] and health behaviour models which theorise processes involving distinct series of changes [21,22]. Interestingly, disparities were noted between sustained campaign awareness and knowledge outcomes. Although it is likely that prolonged campaigns are necessary to achieve widespread positive change, the optimal duration of campaign activity is not yet clear [5]. The findings of this study support the need for planning realistic and targeted outcomes over the short, medium and long term during sustained campaigns. Therefore, it may be useful to include several intermittent time points of outcome evaluation in order to track the patterns of changes as outcomes may change rapidly and may or may not be sustained. Further investigation of how effects persist or vary over time is needed. Future work will compare the effects of this short term campaign with the effects of the national campaign which is run over a longer period of time. Moreover, evaluating the costs and benefits associated with the campaign should be fully investigated. Finally, when considering mechanisms of population change, it might be important to monitor indirect campaign effects such as social discourse or other social networking in the evaluation. This is supported by other studies which have shown that campaigns most potent effects may be through interpersonal communication which may affect both ideation or support [23].

## Competing interests

Time to Change is England's most ambitious programme to end the discrimination faced by people with mental health problems, and improve the nation's wellbeing. Mind and Rethink are leading the programme, funded £16 m from the Big Lottery Fund and £4 m from Comic Relief and evaluated by the Institute of Psychiatry at King's College, London. GT has received grants for research purposes for stigma related research in the last 5 years from Lundbeck UK and from the National Institute for Health Research, and has acted as a consultant to the UK Office of the Chief Scientist.

## Authors' contributions

SEL participated in the conception and design of the study, analysis of data, drafting the article and revising it critically for important intellectual content, and final approval of the version to be published.

JL participated in the acquisition of data, drafting the article or revising it critically for important intellectual content, and final approval of the version to be published.

KL participated in the conception and design of the study, drafting the article or revising it critically for important intellectual content, and final approval of the version to be published.

CH participated in the interpretation of the data, drafting the article or revising it critically for important intellectual content, and final approval of the version to be published.

GT participated in the conception and design of the study, drafting the article or revising it critically for important intellectual content, and final approval of the version to be published.

## Authors' information

SEL and KL are postdoctoral researchers and JL is a researcher in the Health Service and Population Research Department at the Institute of Psychiatry, King's College London. CH is a Senior Lecturer in the Health Service and Population Research Department at the Institute of Psychiatry, King's College London. GT is Professor of Community Psychiatry and Head of the Health Service and Population Research Department at the Institute of Psychiatry, King's College London. He is a Consultant Psychiatrist and Director of Research and Development at the South London and Maudsley NHS Foundation Trust.

## Pre-publication history

The pre-publication history for this paper can be accessed here:

http://www.biomedcentral.com/1471-2458/10/339/prepub
